# The Biotherapeutic Potential of a Novel Probiotic *Kluyveromyces marxianus* Isolated from a Sourdough Starter Against Vaginal *Candida albicans* Strains

**DOI:** 10.3390/jof11020147

**Published:** 2025-02-14

**Authors:** Annalisa Buonanno, Marianna Imparato, Angela Maione, Federica Carraturo, Emilia Galdiero, Marco Guida, Elisabetta de Alteriis

**Affiliations:** 1 Department of Biology, University of Naples “Federico II”, 80126 Naples, Italy; annalisa.buonanno@unina.it (A.B.); marianna.imparato@unina.it (M.I.); angela.maione@unina.it (A.M.); federica.carraturo@unina.it (F.C.); emilia.galdiero@unina.it (E.G.); marco.guida@unina.it (M.G.); 2 BAT Center-Interuniversity Center for Studies on Bioinspired Agro-Environmental Technology, University of Naples “Federico II”, 80055 Portici, Italy

**Keywords:** probiotic yeasts, *Kluyveromyces marxianus*, vulvovaginal candidiasis

## Abstract

There is an increasing interest in yeasts isolated from natural sources to be used as probiotics. *Saccharomyces*-based probiotics have been proposed as a valid alternative to the conventional drug therapy for the prevention and treatment of vulvovaginal candidiasis, also considering the resistance of some *Candida* strains to many antifungals. Here, we isolated from an artisanal sourdough a new yeast strain which was identified as *Kluyveromyces marxianus* and assessed its probiotic and safety properties, which resulted in comparable properties to all those exhibited by the commercial probiotic *Saccharomyces boulardii*. Then, we checked the antagonistic activity of the new isolate against some clinical fluconazole resistant *C. albicans* strains, showing its ability to inhibit filamentation, biofilm formation, and the adhesion of *C. albicans* to vaginal epithelial A-431 cells. Also, *K. marxianus* reduced the cell damage provoked by *C. albicans* and the expression of *SAP2* and *SAP6* genes. On the whole, our results enlarge the spectrum of the beneficial properties of the food-grade yeast *K. marxianus* showing for the first time its biotherapeutic potential against *C. albicans*.

## 1. Introduction

Yeasts are components of the microbiota of various fermented foods and beverages and are largely employed in industry. The discovery of the antagonistic effects of yeasts, especially against other fungal species, has paved the way to focused investigations on their use as probiotics, that is, of their beneficial use for animal and human health [1].

Most conventional probiotics are of a bacterial origin, mainly belonging to the *Lactobacillus* and *Bifidobacterium* genera [2]. More recently, several yeast strains have been reported as promising probiotic candidates as shown by the increasing number of publications over the last decades indicating the relevance of this topic [3]. Some of the differences between yeasts and bacteria, such as the larger size, which can block the attachment of pathogen and spoilage species, the presence of a cell wall containing mannans and beta-glucans, which can positively influence the intestinal microbiome and stimulate innate and acquired immunity, as well as the intrinsic resistance to antibiotics, are considered advantageous for the use of yeasts as probiotics [3].

*Saccharomyces cerevisiae* var. *boulardii*, first isolated in 1920, has been established as a probiotic since 1950, and now it is commercially available throughout the world, mainly used for the treatment of gastrointestinal tract disorders [4]. Later, other *Saccharomyces* and non-*Saccharomyces* yeasts isolated from vegetables, fruits, grains, and traditional fermented foods and beverages have been proposed as probiotics [5,6].

More recently, it has shown how *S. cerevisiae*-based probiotics may also be used for the prevention and treatment of vulvovaginal candidiasis (VVC), a pathology which affects about three-quarters of women during their reproductive age and subjects them to frequent recurrencies [7]. Since the vaginal microbiome plays a relevant role in VVC, a probiotic-based approach could represent a valid alternative to the conventional drug therapy, also considering the resistance of some *Candida* strains to many antifungals and their toxicity for the patients [8].

The effects of a *Saccharomyces*-based treatment against VVC have been investigated both in vitro, and in vivo [9,10,11,12], definitely proving its beneficial protective and therapeutic action.

In this work, we isolated from a sourdough and then characterized a new yeast strain, *Kluyveromyces marxianus*, and assessed its potential probiotic and safety properties, compared to those exhibited by the commercial probiotic *S. boulardii*.

*K. marxianus* isolates from different sources have been reported to have beneficial effects on human health. They include a cholesterol reducing capability [13,14], antiproliferative effects on cancer cells [15,16], immunomodulatory action on Caco-2 cells [17], and also antimicrobial activity against various pathogen species [16,18,19]. In particular, the antifungal activity of different *K. marxianus* strains has been investigated and demonstrated against plant pathogenic fungi [20,21] and the food spoilage molds present in dairy products, so that the yeast has been proposed as a potential biocontrol agent [22]. Instead, so far, the anticandidal activity of this species has not been investigated in detail, being reported only in the case of a *K. marxianus* strain isolated from a French cheese [18].

In a previous work we isolated several *C. albicans* strains from VVC patients and characterized the clinical isolates in terms of the antifungal resistance and biofilm forming ability [23], a characteristic associated to *Candida* virulence.

Therefore, here, aiming at enlarging the number and applications of available probiotic yeasts, we investigated the new *K. marxianus* isolate for its ability to antagonize fluconazole resistant *Candida albicans* vaginal strains, and presented some insights into the mechanisms underlying its protective effect.

## 2. Materials and Methods

### 2.1. Strains and Culture Condition

For the isolation of *K. marxianus*, 10 g of sourdough were suspended in 90 mL of PBS and homogenized with Fisherbrand 850 Homogenizer (Thermo Fisher Scientific, Waltham, MA, USA) for 20 min. The suspended sample was serially diluted in a phosphate buffer saline solution (PBS) and plated onto a Rose Bengal agar (RB, Sigma-Aldrich, St. Louis, MO, USA), supplemented with chloramphenicol. The plates were incubated at 25 °C for 96 h until the typical creamy yeast colonies appeared.

For most experiments, a strain of *S. boulardii* of a commercial origin (Biocodex, Zambon, Italy) was used. It was obtained by dissolving a capsule of the commercial product in PBS and then plating a proper diluted suspension on RB agar plates. After the incubation of the plates at 25 °C for 48 h, colonies appeared, and the typical yeast morphology was checked by microscope observation.

Pre cultures and cultures of both *K. marxianus* and *S. boulardii* were performed in shake flasks containing a YPD (yeast extract 2%, bactopeptone 1%, and dextrose 2%) medium, starting from one single colony, and incubated at 25 °C for 24 h at 200 rpm. The strains of *C. albicans* used were as follows: ATCC 90028, and four clinical fluconazole resistant *C. albicans* strains, namely C7, C14, C17, and C19 isolated from VVC patients [23]. All *C. albicans* strains were cultured in a YPD or RPMI (Thermo Fisher Scientific Inc., Waltham, MA, USA) medium at 37 °C.

In some experiments, to distinguish *C. albicans* from *K. marxianus* colonies, the selective chromogenic medium Brillance Candida Agar Base (Oxoid Ltd., Wade Road, Basingstoke, Hants, UK) was used, which is properly formulated so that *C. albicans* colonies, after incubating agar plates at 37 °C, appear green, whereas *K. marxianus* colonies appear yellow/brown.

### 2.2. Molecular Characterization of the Yeast Isolate

Isolated yeast colonies were sub-cultured in a Dichloran Rose Bengal Chloramphenicol agar (DRBC, Thermo Fisher Scientific Inc., Waltham, MA, USA). The CTAB extraction protocol [24] was employed to extract the fungal total DNA. The extracted DNA was amplified with PCR, targeting the ITS-5.8S rDNA region of the fungal 18S rRNA gene (750 bp amplicon size) [25], employing a MiniAmp™ Thermal Cycler (Thermo Fisher Scientific Inc., Waltham, MA, USA) TECHNE Prime Thermal Cycler, and disposing of a ITS1_f (5′-GGA AGT AAA AGT CGT AAC AAG G-3′ 5′-TCC GTA GGT GAA CCTGCG G-3′) and ITS4_r (5′-TCC TCC GCT TAT TG A TAT GC-3′) primer set (Biofab Research, Rome, Italy). Templates were run on a 1.5% agarose gel, stained with GelRed (BIOTIUM, San Francisco, CA, USA), utilizing a 100 bp DNA ladder as a reference. Sanger Sequencing reactions were performed by an external service (Biofab Research, Rome, Italy); the obtained FASTA sequences were interpreted using an editing tool, Chromas Lite v. 2.6.6 (Technelysium Pty Ltd., South Brisbane, Australia), and compared to the NCBI Sequence Database sequences, using BLASTN ver. 2.2.29 (also referring to GenBank), selecting the highest percentage Identity, with a 98% cut-off and 0.0 e-value.

### 2.3. Probiotic Evaluation of the K. marxianus Isolate

#### 2.3.1. Growth at 37 °C

To test the ability of the *K. marxianus* isolate and the commercial strain of *S. boulardii* to grow at the temperature of the human body, 37 °C, both strains were cultivated at this temperature in shake flasks in YPD for 24 h. Their growth was assessed by OD_590_ determination.

#### 2.3.2. Acid and Bile Salt Tolerance

The acid and bile salt tolerance of *K. marxianus* and *S. boulardii* were determined as already reported [26]. Yeast cells were collected from a 18 h preculture in YPD at 37 °C, washed with PBS, and resuspended in 5 mL PBS plus HCl (pH = 3.0) or PBS plus 0.3% *w/v* bile salts (Sigma Aldrich St. Louis MO, USA), so as to have an initial cell density of 10^8^ cells mL^−1^. After a 4 h incubation at 37 °C, 150 rpm, the CFU mL^−1^ were determined using the standard plate count method, and the percentage of residual viable cells was calculated with respect to the time 0 of incubation (100%).

#### 2.3.3. Auto-Aggregation Capacity

The auto-aggregation capacity of both the *K. marxianus* isolate and *S. boulardii* was determined after 24 h according to Maione et al. [26], by determining the OD_590_ of the yeast suspension before (*OD*_0_) and after (*OD_t_*) incubation. The auto-aggregation (A) percentage was calculated as follows:

$$A=\left[1-\left(\frac{{OD}_{t}}{{OD}_{0}}\right)\right]\times 100$$

#### 2.3.4. Hydrophobicity Assay

The cell biomass was suspended in 5 mL of PBS and the absorbance at 590 nm was measured (*OD*_0_). Then, 3 mL of xylene was added to each sample. After an incubation at 37 °C for 60 min without shaking, the absorbance of the interphase was measured (*OD_f_*) [27]. The hydrophobicity percentage was calculated as follows:

$$H=\left[1-\left(\frac{{OD}_{f}}{{OD}_{0}}\right)\right]\times 100$$

#### 2.3.5. Antioxidant Activity

The radical scavenging activity of *K. marxianus* and *S. boulardii* was evaluated by a 2,2-diphenyl-1-picrylhydrazyl (DPPH) free radical assay [28]. The degree of the discoloration of DPPH indicates the scavenging potential of the strain. A 0.2 mM DPPH solution was freshly prepared in methanol. A total of 800 μL of each strain suspension was mixed with 1 mL of a 0.2 mmol L^−1^. DPPH solution in methanol. The samples were incubated for 30 min in the dark at room temperature with agitation and then centrifuged at 2000 rpm for 2 min. The supernatant absorbance was measured at 517 nm. In parallel, ascorbic acid was used as a positive control. The scavenging ability (SA) of the strain was calculated using the formula:

$$SA=[1-\frac{{OD}_{517}\left(sample\right)}{{OD}_{517}\left(blank\right)}]\times 100$$

#### 2.3.6. Assessment of Biofilm Formation

This was performed as described previously [29] and detected using the crystal violet staining method for the total biofilm biomass determination, measured by OD_570_ after the crystal violet solubilization with acetic acid [30].

#### 2.3.7. A-431 Cells

The human epithelial A-431 cell line derived from a vaginal epithelial squamous cell carcinoma was used. The cell line was purchased from DSZM (Braunschweig, Germany). The cells were cultured in Dulbecco’s modified Eagle’s medium (DMEM; Sigma Aldrich Co., St. Louis, MO, USA), supplemented with L-glutamine (2 mM) (Sigma Aldrich Co., St. Louis, MO, USA), 1% *w*/*v* penicillin–streptomycin (Sigma Aldrich Co., St. Louis, MO, USA), and a heat-inactivated Fetal Bovine Serum (FBS), at 10% *w*/*v* (Sigma Aldrich Co., St. Louis, MO, USA); specifically, the cell line was kept in culture by-passages in fresh medium twice a week and incubated at 37 °C and 5% CO_2_.

#### 2.3.8. Adhesion to A-431 Cells

To allow for the adhesion to human cells, *K. marxianus* and *S. boulardii* cells at a density of 10^6^ cells mL^−1^ were added to the cell monolayer in a 48 well multiwell plate and incubated for 2 h in DMEM at 37 °C. Then, the wells were washed with PBS, and a 100 μL di tripsin/EDTA solution (Sigma Aldrich Co., St. Louis, MO, USA) was added. After 5–6 min of incubation, 900 μL di DMEM was added for trypsin inactivation. The content of the wells was serially diluted in PBS and plated onto a chloramphenicol RB agar for the yeast cell count.

Also, the invasiveness of *K. marxianus* and *S. boulardii* into A-431 cells was determined, following the above-described protocol, but, before the trypsin treatment, the infected A-431 cells were incubated with 0.5 mg mL^−1^ amphotericin B for 1 h, to eliminate the adhered cells on the surface and to allow us to count only the internalized yeast cells.

### 2.4. Safety Assessment of the K. marxianus Isolate

#### 2.4.1. Haemolytic Activity

The haemolytic activity of the isolate and, in parallel, of *S. boulardii,* was carried out according to the method described by Shen et al. 2022 [31], by inoculating the strains on blood agar plates (Oxoid Ltd., Basingstoke, UK) containing 5% defibrinated sheep blood for 48 h of an incubation at 37 °C in order to detect patterns of haemolysis. Next, cultured plates were observed to assess a haemolytic zone, and a clear zone of hydrolysis around the colonies was considered as a positive result (β-haemolysis).

#### 2.4.2. Antibiotic and Antifungal Resistance

Antimicrobial resistance was screened by the agar disk diffusion method as described by Turchi et al. 2013 [32], in accordance with the Clinical and Laboratory Standards (M44, M45). The isolated yeast strain was tested for resistance against four antifungal compounds. The molecules tested were fluconazole (10 μg FL), caspofungin (2 μg CSF), ketoconazole (30 μg KET), and amphotericin B (2 μg Amph B). After a 24–48 h incubation at 30 °C, the diameter of the inhibition zone was measured and the strains were considered susceptible (S), intermediate (I), or resistant (R).

#### 2.4.3. Galleria Mellonella Survival Assay

To evaluate the safety of the *K. marxianus* isolate in vivo, larvae of *G. mellonella* were used. For each assay, 20 healthy larvae of a similar size, were selected and injected with 10 μL of the *K. marxianus* suspension in PBS through the last left pro-leg, at a density corresponding to 10^4^, 10^5^, 10^6^, and 10^7^ yeast cells/larva. A similar procedure was performed for the commercial *S. boulardii*. Intact larvae or the injected with PBS alone were used as controls. All samples were incubated at 37 °C for 120 h. Every 24 h, the number of viable larvae was registered, and the survival percentage was calculated. Larvae were considered dead when they displayed no response to touch.

### 2.5. Anticandidal Activity of the K. marxianus Isolate

#### 2.5.1. Co-Aggregation of K. marxianus with C. albicans

The co-aggregation of the *K. marxianus* isolate with *C. albicans*, was determined as previously described [26]. Briefly, 4 mL of a *K. marxianus* overnight culture and 4 mL of a *C. albicans* overnight culture were mixed, vortexed for 10 s, and incubated for 5 and 24 h at 37 °C. Each control tube contained 4 mL of each single suspension. The absorbance (*OD*) of the mixed suspension was then measured at 590 nm (*OD_mix_*) and compared with those of the control tubes containing *K. marxianus* (*OD_strain_*) and the pathogen (*OD_pathogen_*) after incubation. The percentage of co-aggregation (*C*) was calculated as follows:

$$C=\left[1-{OD}_{mix}\times \frac{\left({OD}_{strain}+{OD}_{pathogen}\right)}{2}\right]\times 100$$

#### 2.5.2. Exclusion Test

The ability of pre-established *K. marxianus* biofilms to prevent the adhesion and biofilm formation of *C. albicans* on polystyrene microplates was evaluated performing an “exclusion test” according to Cisneros et al. 2021 [33], with some modifications. *K. marxianus* cells derived from overnight cultures in YPD were adjusted to 10^6^ CFU mL^−1^ and seeded in individual wells of a 96-well multiplate. Microplates were first incubated at 37 °C for 24 h to allow cells to attach to the surface of the wells. Thereafter, supernatants were discarded, and a suspension of each *C. albicans* strain in RPMI (10^6^ CFU mL^−1^) was added to each well containing the *K. marxianus* pre-formed biofilm. Microplates were incubated at 37 °C for 24 h. Then, wells were carefully washed twice with PBS and then individually scraped to remove all the biofilm biomass. Cells recovered from each well were suspended, diluted in PBS, and then plated on Brillance Candida Agar Base plates to distinguish and determine the number of viable cells of *C. albicans* and *K. marxianus*, respectively. The plates were incubated at 37 °C for 48 h. Then, for each strain, the number of colonies on the plates was determined. The results were expressed as Log_10_ (CFU well^−1^). The biofilm formation of *C. albicans* strains was used as control.

#### 2.5.3. Germ Tube Test in the Presence of *K. marxianus*

The ability of *C. albicans* to form germ tubes was assessed using the germ tube test according to Pericolini et al. [9]. Briefly, an isolated colony of *C. albicans* of each strain (ATCC 90028, C7, C14, C17, and C19) taken from a 24 h YPD agar plate, was inoculated into a total volume of 0.5 mL of RPMI plus 10% Fetal Bovine Serum (Fisher Thermo Scientific, Cleveland, OH, USA) in the well of a 24-well multiplate and incubated for 4 h at 37 °C, in the presence or not of 10^8^ cells mL^−1^ of *K. marxianus* resuspended in a RPMI medium. After incubation, the wells were examined with a JuLI™ Stage inverted microscope (NanoEntek, Seoul, Korea) at a 20× magnification.

#### 2.5.4. The Adhesion of *C. albicans* to A-431 Cells in the Presence of *K. marxianus*

To investigate the probiotic effects of *K. marxianus* on the *Candida* adhesion to A-431 cells, we followed the protocol by Kunyet et al. [11] with some modifications. The assays of adhesion on human cells were performed under pre-inoculation, co-inoculation, and post-inoculation conditions in 48-well microplates where A-431 cells in DMEM were at 80% confluency. In all cases, the density of the probiotic yeast and of each *C. albicans* strain (ATCC 90028, C7, C14, C17, and C19) was 10^8^ cells mL^−1^ and 10^7^ cells mL^−1^, respectively.

In the pre-inoculation condition, *K. marxianus* was inoculated into wells for 2 h at 37 °C. Then, the *Candida* strain was introduced and incubated for an additional 2 h with mild shaking (90 rpm). In the co-inoculation condition, the probiotic strain was co-inoculated with the *Candida* strain and incubated for 2 h. In the post-inoculation condition, the *Candida* strain was inoculated for 2 h before the inoculation of *K. marxianus* for a further 2 h.

At the end of incubation, the wells were washed with PBS, and their content was serially diluted in PBS. The dilutions were plated on a Brillance Candida Agar Base to distinguish *C. albicans* from *K. marxianus* colonies and count the residual *C. albicans* colonies. The results were expressed as a percentage inhibition of the adhesion, with respect to the controls performed in the same condition without the inoculation of the probiotic.

#### 2.5.5. Cell Damage: LDH Determination

A-431 Cells were then incubated in the presence or absence of *K. marxianus* (OD_590_ = 5) for 2 h at 37 °C plus 5% CO_2_, extensively washed with PBS, and then incubated with *C. albicans* ATCC 90028 or *C. albicans* C19 (1 × 10^6^ mL^−1^) for 18 h at 37 °C plus 5% CO_2_.

After incubation, the epithelial cell damage was determined by the release of lactate dehydrogenase (LDH) into the surrounding medium. Therefore, the medium was collected and LDH was measured spectrophotometrically at 492 nm using a Lactase Dehydrogenase Activity Assay kit (Sigma-Aldrich, St. Louis, MO, USA). The percentage cell damage of the A-431 infected with *C. albicans* was calculated as follows:

$$Cell\;damage=\frac{\left(LDH\;activity\;of\;treated\;cells-spontaneuous\;LDH\;activity\right)}{\left(maximum\;LDH\;activity-spontaneous\;LDH\;activity\right)}\times 100$$

and compared to the 100% *C. albicans* damage induced in each cell type [34]. The lysis buffer was used as a positive control to determine the maximum LDH activity.

#### 2.5.6. Quantitative Analysis of *SAP2* and *SAP6* Gene Expression

To check the expression of the *SAP2* and *SAP6* genes of *C. albicans* in the presence of *K. marxianus*, aliquots of *C. albicans* cells of ATCC 90028 and C19 strains (10^6^ mL^−1^) were incubated in a YPD medium or a YPD medium plus 1% of the bovine serum albumin (BSA), for 24 h at 37 °C under agitation (150 rpm) in the presence or absence of *K. marxianus* cells (OD_.590_ = 5.0) [9]. Briefly, the total RNA was extracted using the Direct-zolTM RNA Miniprep Plus Kit (ZYMO RESEARCH, Irvine, CA, USA). Then, 1 μg of the RNA was reverse transcribed to cDNA (Bio-Rad, Milan, Italy) and analyzed by a quantitative PCR run in a AriaMx Real-Time PCR instrument (Agilent Technologies, Inc., Milan, Italy) according to the manufacturer’s instructions. For the real-time PCR reaction, 100 ng of cDNA was used. The PCR System used was as follows: 95 °C for 10 min, one cycle for cDNA denaturation; 95 °C for 15 s and 60 °C for 1 min, 40 cycles for amplification; 95 °C for 15 s, one cycle for final elongation; and one cycle for melting curve analysis (from 60 °C to 95 °C) to verify the presence of a single product. *C. albicans SAP2* and *SAP6* genes were detected by using the primers previously reported [35]. The expression levels of genes were evaluated using the 2^−∆∆CT^ method, where Ct was the average threshold cycle number from three independent experiments. Data were presented as the fold change in the gene expression normalized to the *ACT1* gene as an internal control [36,37].

### 2.6. Statistical Analyses

GraphPad Prism Software (version 8.02 for Windows, GraphPad Software, La Jolla, CA, USA, www.graphpad.com, accessed on 14 February 2024) was used for data analysis. All data are shown as mean ± standard deviation (SD) and were derived from two or three independent experiments. One-way or two-way analysis of the variance (ANOVA) followed by Tukey’s or Sidak’s test was used for the comparison test. The Kaplan–Meier method and log-rank (Mantel–Cox) test were used to plot *G. mellonella* survival curves.

## 3. Results

### 3.1. Isolation and Molecular Characterization of K. marxianus Isolate

The yeast isolate from sourdough was subjected to molecular identification. Results of the Sanger Sequencing of the yeast colonies is reported in Table 1. The isolate *Kluyveromyces marxianus* was discovered.

### 3.2. Probiotic Properties of the K. marxianus Isolate

Some characteristics of the *K. marxianus* isolate were determined to assess the potential probiotic properties of the strain. They were as follows: the ability to grow at 37 °C, a tolerance to gastric and bile acids, auto-aggregation, hydrophobicity, antioxidant power, biofilm forming capacity, and the adhesion to epithelial cell lines. In parallel, the same tests were conducted with the commercial *S. boulardii* (Biocodex) strain.

Overall, these tests are indicative of the adaptation of the isolate to the human body (growth at 37 °C, and resistance to acid and bile salts), with potential beneficial effects (antioxidant power) and the ability to adhere and colonize the epithelial mucosal membranes (hydrophobicity, auto-aggregation, biofilm forming ability, and adhesion).

The results of the tests are reported in Table 2, where a score from 0 (minimum) to 2 (maximum) was given to the result of each test response for both the *K. marxianus* isolate and *S. boulardii*.

**Figure 1:**
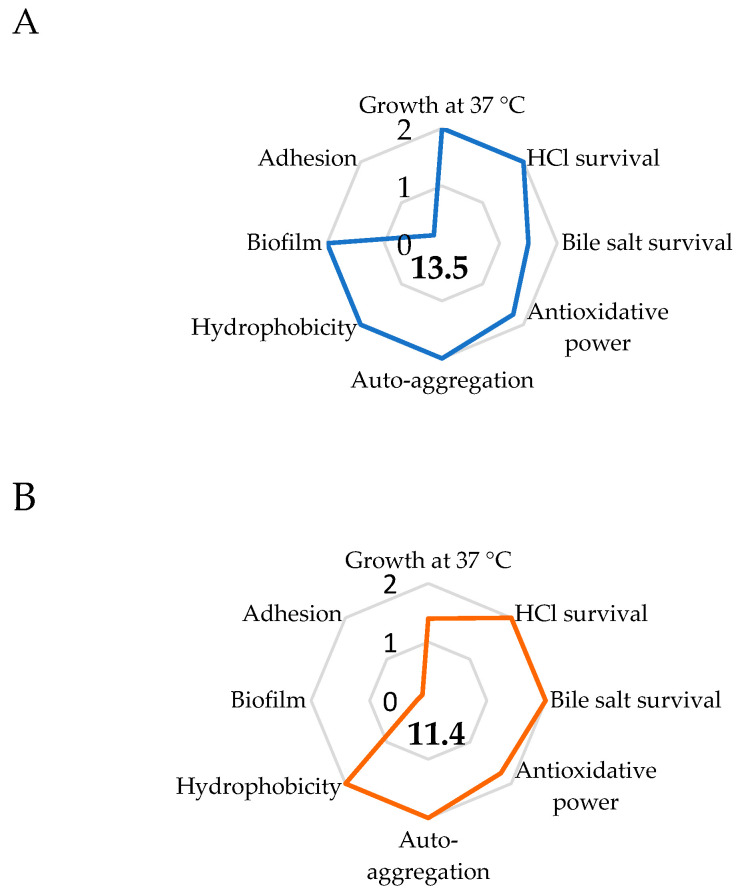
Radar diagrams of the probiotic properties (see Table 1) of *K. marxianus* (**A**) and *S. boulardii* (**B**). The total score of the strain is reported in the center of the diagram.

For a better visualization of the overall probiotic characteristics of each strain, the indicators in Table 2 were reported in radar diagrams (Figure 1).

The *K. marxianus* isolate showed very good probiotic properties (total score 13.5), even better than the commercial *S. boulardii* strain (total score 11.4). Most characteristics resulted similarly between the two yeasts, but the *K. marxianus* isolate showed a better adaptation to the body temperature (37 °C), a much better ability to form biofilm in vitro, and a higher adhesion to A-431 cells than *S. boulardii (*Table 2 and Figure 1*)*. Though the adhesion to A-431 cells was not so high, it is worth noting that the invasiveness of both the strains into the A-431 cells was absent.

### 3.3. Safety Assessment

The safety of the *K. marxianus* isolate was assessed in vitro by testing both its haemolytic activity on blood agar plates and its resistance to antibiotics and antifungals.

**Figure 2:**
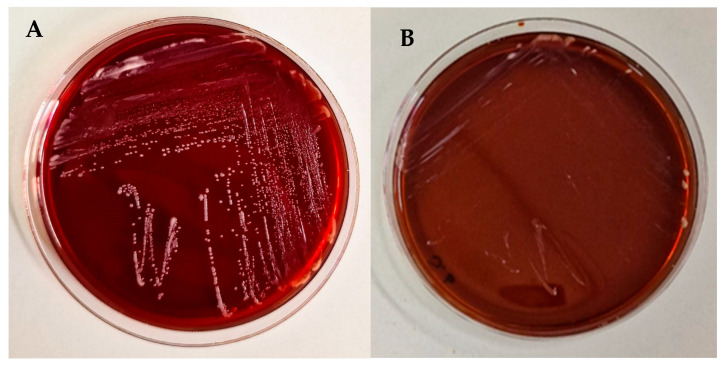
Blood agar plates showing the absence of haemolytic activity for *K. marxianus* (**A**) and *S. boulardii* (**B**).

As shown in Figure 2, no haemolytic activity was shown by the *K. marxianus* isolate, as well as by the *S. boulardii* strain.

In regards to the resistance to antimicrobials, the yeast isolate was susceptible to the tested antifungals. A similar response was obtained by *S. boulardii* (Table 3).

**Figure 3:**
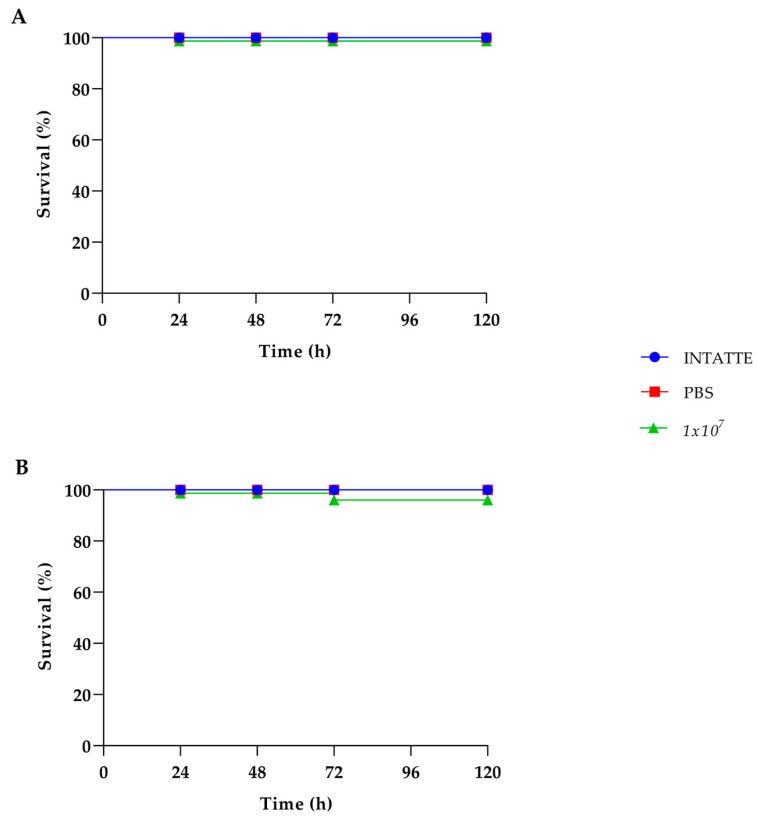
Survival curves of the *G. mellonella* larvae injected with *K. marxianus* (**A**) and *S. boulardii* (**B**) at a density of 10^7^ CFU per larva.

To assess the safety in vivo of the *K. marxianus* isolate, we used larvae of *Galleria mellonella*. The larvae survival after injection of the yeast strain at different final cell densities (from 10^4^ to 10^7^ yeast cells per larva) was determined. In parallel, the same test was carried out with the *S. boulardii* strain. No effect of the yeast injection was observed in the case of the *K. marxianus* isolate, since the larvae survival remained 100% up to 120 h incubation, likewise to what was observed in the case of the commercial probiotic *S. boulardii* (Figure 3).

### 3.4. Activity of K. marxianus Against C. albicans

The co-aggregation of a probiotic with a pathogen is indicative of its capacity to prevent the subsequent adhesion and colonization; therefore, we first tested the ability of the *K. marxianus* isolate to co-aggregate with *C. albicans*. The percentages of co-aggregation were significant, being about 50% with both ATCC 90028 and the C19 strain after a 5 h incubation and this increased up to 75% for both strains after 24 h.

**Figure 4:**
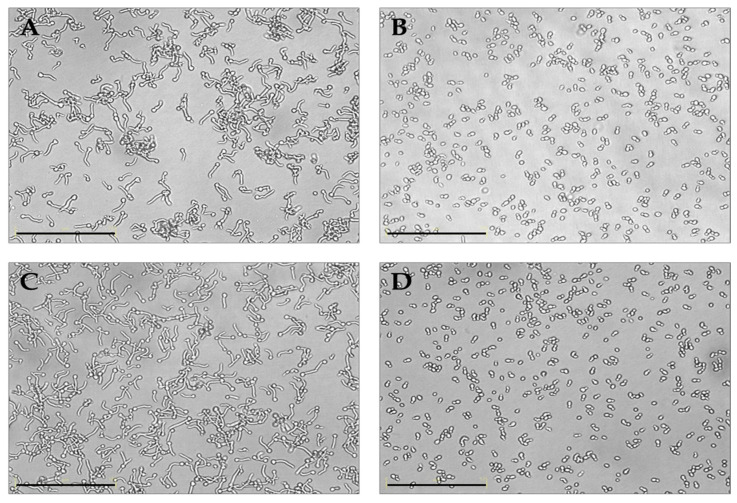
Inhibition of the filamentation of *C. albicans* ATTC 90028 (**A**,**B**) and *C. albicans* C19 (**C**,**D**) in the presence of *K. marxianus* (**B**,**D**). In panels (**A**,**C**), controls are shown. The bar corresponds to 125 μm.

Then, we wanted to assess the effect of the putative probiotic *K. marxianus* on the filamentation of *C. albicans* strains, considering that filamentation is a key virulence factor for *Candida* species and is positively correlated with adhesion and biofilm formation. In Figure 4, the results of a germ test of *C. albicans* ATCC and *C. albicans* C19 in the presence of *K. marxianus* are shown. A clear, full inhibition of the germ tube formation was observed for both of the strains. Similar results were obtained with all the other *C. albicans* vaginal strains.

**Figure 5:**
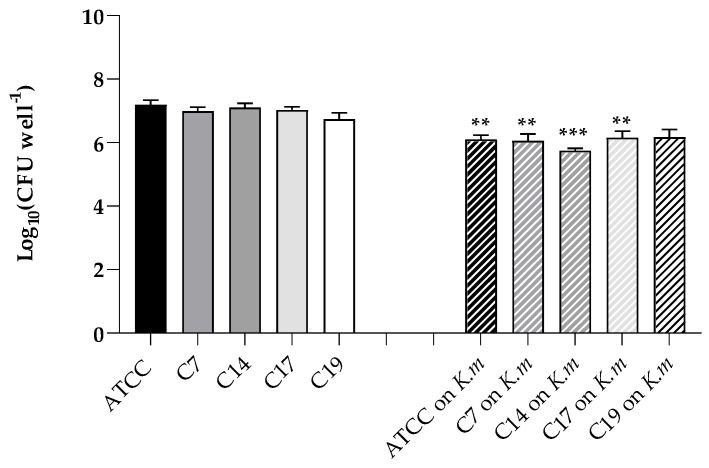
Reduction of the biofilm formation (Log_10_ CFU well^−1^) in polystyrene microwells of the *C. albicans* ATCC 90028, C7, C14, C17, and C19 strains in the presence of a preformed *K. marxianus* biofilm. Data reported are the means of three independent experiments ± SDs; statistical significance is indicated by ** *p* < 0.01; *** *p* < 0.001 (Tukey’s test).

As previously reported [23], all the *C. albicans* strains used in this work were able to form biofilms in vitro on polystyrene microplates. Here, we assessed the ability of a pre-established biofilm of the *K. marxianus* isolate to prevent such a biofilm formation. So, the formation of the biofilms of *C. albicans* ATTC 9028 and the clinical strains were evaluated in the so called “exclusion test”. The results clearly indicated that surface colonization and ability to form biofilms was significantly reduced in the presence of a pre-formed *K. marxianus* biofilm for all the *C. albicans* strains examined (Figure 5).

**Figure 6:**
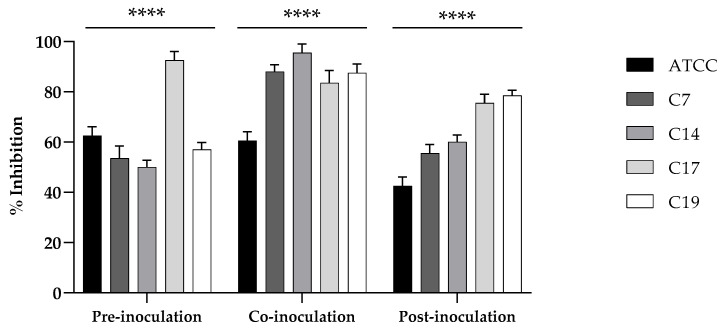
Inhibition of the adhesion of *C. albicans* ATCC 90028, C7, C14, C17, and C19 strains on A-431 cells in conditions of pre-inoculation, co-inoculation, and post-inoculation with the *K. marxianus* isolate. Data reported are the means of three independent experiments ± SDs. The statistical significance in respect to the corresponding controls is indicated by **** *p* < 0.0001 (Tukey’s test).

Also, we tested the effect of the putative probiotic on the adhesion of *Candida* strains on A-431 cells, investigating different conditions of treatment: pre-inoculation, where the probiotic yeast was added to the cell monolayer prior to application of the *Candida* strains; co-inoculation, where the live probiotic yeast and *Candida* strains were simultaneously applied; and post-inoculation, where the probiotic yeast treatment was performed after the application of *Candida* strains. In the three conditions and for all of the strains examined, the reduction of adhesion was clear (Figure 6), and resulted very high (90%) for the strains C7, C14, and C19 in the condition of co-inoculation.

**Figure 7:**
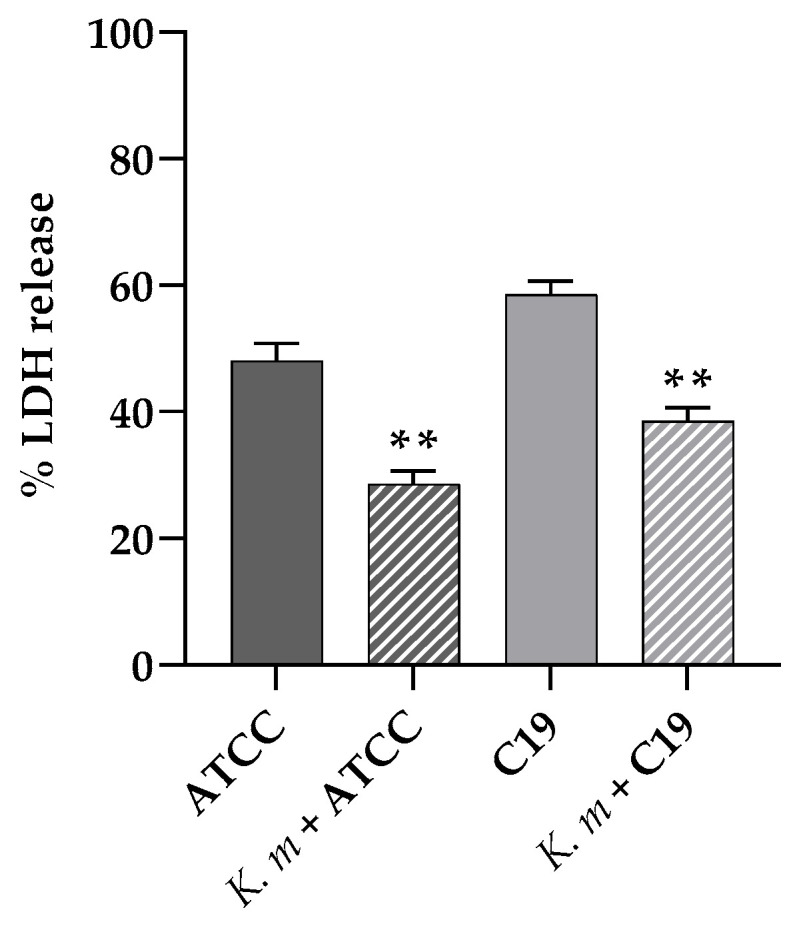
Cell damage evaluated by the LDH release from A-431 cells infected with the *C. albicans* ATCC 90028 or C19 strain and the effect of the probiotic *K. marxianus*. Data reported are the means of three independent experiments ± SDs; statistical significance is indicated by ** *p* < 0.01 (Sidak’s test).

The possible protective effect of *K. marxianus* against the damage provoked by the *C. albicans* infection in A-431 cells was also ascertained, determining the LDH release from the cell line preincubated with the *K. marxianus* isolate and then infected with *C. albicans* ATCC 90028 or *C. albicans* C19 for 18 h. The results showed a clear prevention of the cell damage provoked by the *C. albicans* strains in the presence of the probiotic yeast (Figure 7). It is worth noting that the *K. marxianus* isolate did not exert any significative cytotoxic effect on A-431 cells, since the LDH released from the cells treated with the probiotic alone was comparable with the spontaneous release of LDH.

**Figure 8:**
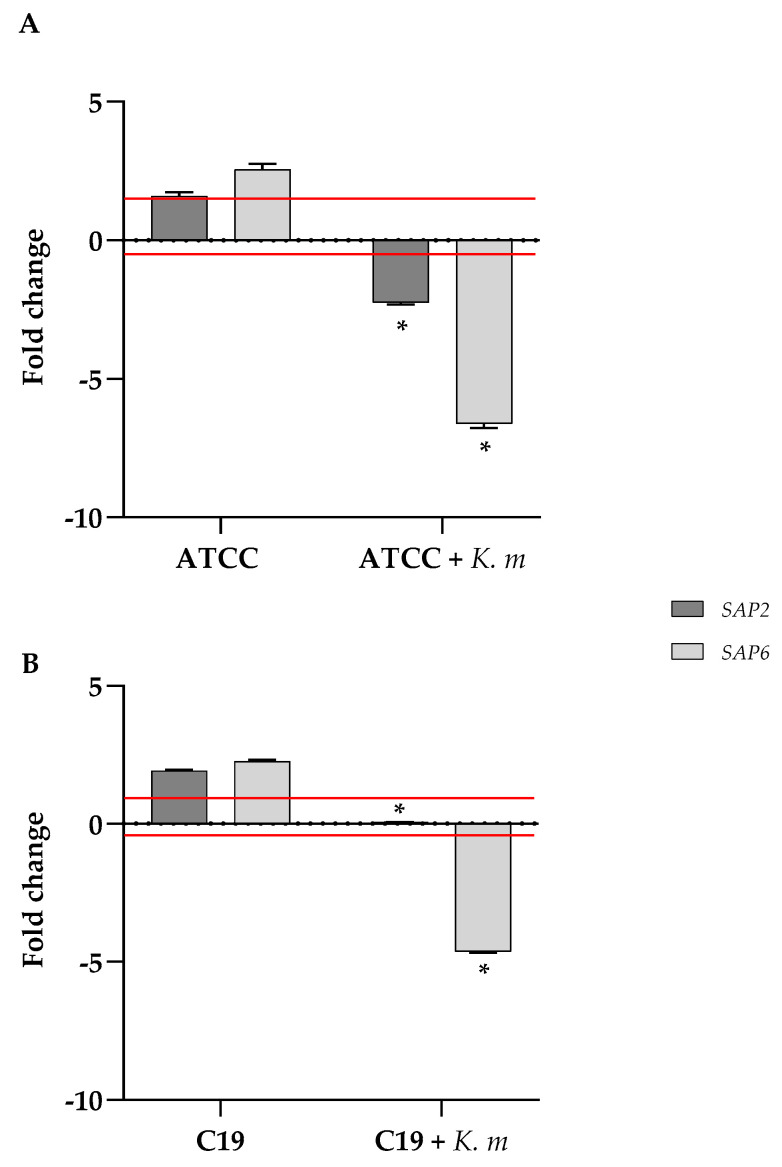
Quantitative analysis of the *SAP2* and *SAP6* genes expression in *C. albicans* ATTC 90028 (**A**) and *C. albicans* C19 (**B**) strains, incubated in a YPD medium plus 1% BSA with or without *K. marxianus*. cDNA quantities were reported as fold changes relative to the *Candida* strain alone. Data reported are the means of three independent experiments ± SDs. Red lines represent fold change thresholds of 1 and −0,5. Statistical significance is indicated by * *p* < 0.1 (Sidak’s test).

Since aspartyl proteases exert a main contribution to the virulence and invasiveness of *C. albicans*, we checked the expression of two of the genes (*SAP2* and *SAP6*) encoding for such proteins when *C. albicans* was co-incubated with the putative probiotic *K. marxianus* isolate. The results of the RTqPCR (Figure 8) clearly show that in the presence of the isolate, the expression of both *SAP2* and *SAP6* resulted greatly reduced both in the ATCC and the clinical C19 strain.

## 4. Discussion

In recent years, the interest towards the probiotic potential of non-conventional yeasts has greatly increased. Non-*Saccharomyces* yeasts mainly belonging to the genera of *Pichia, Debaryomyces, Wickerhamomyces, Zygosaccharomyces*, and *Kluyveromyces* have been isolated from different sources and tested as probiotics, as an alternative to the well-established commercial species *S. cerevisiae* var. *boulardii* [6,38].

The food-grade yeast *K. marxianus* is characterized by a rapid growth rate, easy cultivation, thermotolerance, a versatile metabolism, and the ability to secrete lytic enzymes, which make this species particularly suitable for many food and biotechnological applications [39,40].

As specifically regards the probiotic properties of *K. marxianus*, they have been ascertained since 2011, when it was shown that the yeast strain isolated from a dairy product [17] was able to adhere to human enterocyte-like Caco-2 cells and modulate colon microbiota, increasing the bifidobacterial concentration, and inducing the formation of higher amounts of the short-chain carboxylic acids acetate and propionate. The strain was *K. marxianus* B0399, which was subsequently introduced in the market as a new generation of probiotic lactic yeast (Turval B0399^®^).

Later on, other authors have reported several other beneficial effects of *K. marxianus* mainly isolated from dairy products [41]. Very recently, Nag et al. [42] observed in vitro that this yeast improved insulin sensitivity and reduced fat storage in fat cells, suggesting benefits for type 2 diabetes and obesity. Moreover, *K. marxianus* produce high value-added bioingredients (oligo-saccharides, -nucleotides, -peptides) that, used as prebiotics, have been revealed to increase the intestinal microbiota or if added to foods, enhance flavors, stabilize food emulsions, act as immunopotentiators, have hypocholesterolemic action, and promote protection against bacterial infections [43].

In this work, we isolated a *K. marxianus* strain from a sourdough used in an artisanal bakery as a starter for the leaving of bread. This practice, which has recently undergone a revival also in industrial bakeries, exploits the spontaneous fermentation of yeasts and lactobacilli present in the sourdough, and confers the final product a distinctive taste and aroma.

Aiming at proposing the new *K. marxianus* isolate as a potential probiotic, we performed an initial in vitro screening of the properties necessary to adapt to the human body with beneficial effects. The tests were done in parallel with a reference probiotic strain, the commercial *S. boulardii* (Biocodex). To comprehensively evaluate and visualize the results, radar charts were used [31], which clearly indicate that the new strain has excellent probiotic properties, a result which is in agreement with those reported for other *K. marxianus* isolates [22]. The comparison showed that *K. marxianus* better adapts to the body temperature and forms thicker biofilm in vitro. A good biofilm ability on abiotic surfaces has been reported in the case of several *K. marxianus* strains from different dairy products, and it is considered an essential trait for the outcome of the final product [44]. In regards to the adhesion to vaginal epithelial cells, it resulted higher than that of *S. boulardii* on the same cells, but not so high (10%). Many different values of the adhesion to Caco-2 cells have been reported for *K. marxianus*, varying from 8 to 19% depending on the strain [45].

The adhesion of probiotics to epithelial cells is generally viewed as a prerequisite for their colonization, which may constitute a physical barrier against the adhesion of pathogens to the mucosa. However, also low-adhering *S. cerevisiae* strains have shown to display protective effects towards bacterial infections, via a reduction of the intestinal pro-inflammatory response [16]. For some authors [46,47], a good adhesion of a probiotic strain is even considered an undesirable virulence factor, because it precedes the possible invasion of the strain into the human cells. In our case, it is worth noting that the invasion of *K. marxianus* into A-431 cells was totally absent.

A safety evaluation was conducted to confirm the non-pathogenic nature of the *K. marxianus* isolate: the yeast resulted in being non-haemolytic and susceptible to the tested common antifungals.

Due to their simplicity of use and low cost, the larvae of *G. mellonella* represent an optimal model to evaluate the virulence of fungi and other microbial species [48]. Then, to assess the safety of the *K. marxianus* isolate also in vivo, we used the *G. mellonella* model. Indeed, *G. mellonella* larvae have been established as an animal model alternative to mammals to test the pathogenicity of fungal species since 2000 [49], and more recently has been used for the study of the safety of probiotic putative strains in vivo [26,50]. Our results clearly showed that the survival of the *Galleria* larvae was not affected at all by the injection with *K. marxianus*, even at the highest density tested, similarly to what is observed with *S. boulardii*. So, the in vivo tests unequivocally assessed the safety of the new *K. marxianus* isolate.

*C. albicans* is the agent of the vast majority of VVC [51], with an increasing rate of episodes associated to fluconazole resistant strains [52]. In the perspective of a possible use of *K. marxianus* as a novel probiotic for both the prevention and therapy of VVC, we analyzed the antagonistic activity of the yeast isolate against some vaginal strains of *C. albicans*.

Our results clearly showed the co-aggregation ability of *K. marxianus* with *C. albicans* and a full inhibition of the germ tube formation upon treatment with the live yeast, a result in agreement with other authors who found the same effects with *Saccharomyces* strains [9,10]. It is well known that the polymorphic *C. albicans* may shift from the yeast-like morphology to germ tube, pseudo-hyphae, and hyphae. Such a filamentation is pivotal for biofilm formation and epithelial invasion, representing a well-known virulence factor for *Candida* [53].

Further, and in accordance with this result, we demonstrated a reduction of the biofilm formation in vitro for all the tested *C. albicans* strains in the presence of the *K marxianus* preformed biofilm. Such an “exclusion effect” [33] is indicative of a clear antagonism between the putative probiotic and *Candida*, due to a physical block for the pathogen colonization on the abiotic surface, without excluding the contribution of other mechanisms of action.

The adhesion of *Candida* to the epithelial vaginal cells is certainly mandatory for invasiveness and pathogenesis, so we also investigated the effect of *K. marxianus* on the *Candida* adhesion to A-431 cells. Its results were significantly impaired by the presence of the live putative probiotic, which showed its efficacy in both the inhibition of the adhesion and displacement of *Candida* cells, suggesting its potential use in both preventing and ameliorating *Candida* infections. These results are in agreement with those obtained by Kunyet et al. [11] with *Saccharomyces* strains able to hinder the *Candida* adhesion both on polystyrene surfaces and A-431 cell lines.

Indeed, the presence of a *K. marxianus* isolate protected the epithelial cells from the damage induced by *C. albicans*, as shown by the lesser amount of LDH released from the A-431 cells pretreated with the probiotic strain and then infected with the pathogen.

The secretion of aspartyl proteases encoded by *SAP* genes plays an essential role in the development of the pathogenesis of *C. albicans*, being involved in the shift of the yeast to a hyphal morphology which is determinant for invasiveness [54]. Consistently with the observed inhibition of the germ tube formation, we also demonstrated the ability of the probiotic isolate to reduce the expression of *SAP2* and *SAP6* genes in *Candida* under conditions of serum-induced hyphal differentiation. This result is in agreement with what is already observed for other yeasts proposed as anticandidal probiotic agents [9,10].

Apparently, *K. marxianus* is able not only to protect the epithelial cells from adhesion via a mechanical hindrance to *Candida* colonization, but also to affect some virulence factors such as hyphal differentiation and the secretion of aspartyl proteases, presumably due to products of its metabolism and/or competition for those serum components inducing *Candida* hyphal morphogenesis. Further investigations are necessary to shed light on the details of *Kluyveromyces/Candida* interactions.

In conclusion, our results enlarge the spectrum of the beneficial properties of the food-grade yeast *K. marxianus*. The assessment of its probiotic properties such as the adhesion to epithelial vaginal cell lines and the ability to antagonize *C. albicans*, make it a promising biotherapeutic agent in the prevention and treatment of VVC.

## Figures and Tables

**Table 1:** Results of the Sanger Sequencing performed on yeast colonies isolated from sourdough. Obtained FASTA Sequences were compared to NCBI Sequence Database sequences, using BLASTN ver. 2.2.29.

Identified Microorganism	Max Score	Total Score	Query Cover	e-Value	% Identity	Accession N.
*Kluyveromyces marxianus strain JYC2557*	1223	1223	99%	0.0	99.55%	MK044038.1

**Table 2:** Results of the tests on the probiotic properties of *K. marxianus* (Km) and *S. boulardii* (Sb), and the scores attributed to each test (in italics).

	Growth at 37 °C	HCl Survival	Bile Salt Survival	Antioxidative Power	Auto- Aggregation	Hydrophobicity	Biofilm	Adhesion	Total Score
	(OD_590_)	(%)	(%)	(%)	(%)	(%)	(OD_570_)	(%)	
Km	6.86	100	73	78.5	100	90	4.5	10	
	2	2	1.5	1.75	2	2	2	0.2	13.5
Sb	4.93	100	98	77	100	81	0.3	2.35	
	1.4	2	2	1.75	2	2	0.2	0.05	11.4

**Table 3:** The antifungal susceptibly to fluconazole (FLC), caspofungin (CSF), ketoconazole (KET), and amphotericin B (amphB) of the *K. marxianus* isolate and commercial *S. boulardii*. The strains were considered susceptible (S), intermediate (I), or resistant (R).

	FLC	CSF	KET	AmphB
*K. marxianus*	S	S	S	S
*S. boulardii*	S	S	S	S

## Data Availability

The original contributions presented in the study are included in the article. Further inquiries can be directed to the corresponding author.
